# Novel glucose-lowering agents that benefit diabetic foot: icing on the cake

**DOI:** 10.3389/fendo.2025.1581403

**Published:** 2025-07-02

**Authors:** Qin Li, Shiyan Yu, Shunli Rui, Xiaobu Lin, Yi Yuan, David G. Armstrong, Yi Xu, Wuquan Deng

**Affiliations:** ^1^ Department of Endocrinology and Diabetic Foot Center, Chongqing University Central Hospital, Chongqing Emergency Medical Centre, School of Medicine, Chongqing University, Chongqing, China; ^2^ Department of Surgery, Keck School of Medicine of University of Southern California, Los Angeles, CA, United States; ^3^ Chongqing Liang Jiang New Area Traditional Chinese Medicine Hospital, Chongqing, China; ^4^ Department of Population Health Sciences, King's College London, London, United Kingdom

**Keywords:** diabetic foot, novel glucose-lowering agents, dipeptidyl peptidase-4 inhibitors, glucagon-like peptide-1 receptor agonists, sodium-glucose cotransporter-2 inhibitors

## Abstract

The prevalence of diabetes mellitus is increasing and is linked to several complications, including diabetic foot. Novel glucose-lowering agents are sought that also have beneficial effects in reducing diabetic complications. Among the novel glucose-lowering agents demonstrating clinical promise, three classes stand out: dipeptidyl peptidase-4 inhibitors (DPP-4is), glucagon-like peptide-1 receptor agonists (GLP-1RAs), and sodium-glucose cotransporter-2 inhibitors (SGLT2is). Some of these agents provide cardiovascular and kidney benefits, and there is evidence suggesting they also offer protective effects against diabetic foot complications. In this review, we summarize the preclinical and clinical evidence proof these three glucose-lowering agents for diabetic foot, highlighting their potential in enhancing diabetic wound healing and limb preservation. In conclusion, existing available trials have shown that certain DPP-4is and GLP-1RAs possess protective effects against diabetic foot conditions. However, SGLT2is have not demonstrated a significant protective effect. We encourage larger-scale studies on the protective effects of these three types of drugs for diabetic foot to guide physicians in providing personalized treatment strategies, achieving blood glucose targets, and promoting the healing of chronic wounds in patients.

## 1 Introduction

Type 2 diabetes mellitus (T2DM) constitutes a significant global health challenge, affecting an estimated 451 million people around the world (1). As a chronic complication of diabetes, diabetic foot is characterized by structural and functional disturbances in the foot. These problems arise from changes in the peripheral blood vessels and nerves of the lower extremities (2, 3). It involves numerous risk factors and presents complicated mechanisms alongside minor clinical manifestations. Between 19% and 34% of diabetic individuals are prone to developing diabetic foot (4). The substantial burden, economic costs, and mortality rates linked to diabetic foot are comparable to those of cancer (5). Diabetic foot-related problems result in prolonged hospitalization, significant economic burdens on healthcare, and a diminished quality of life (6). Furthermore, diabetic foot is recognized as the predominant cause of non-traumatic lower extremity amputations globally (7). Standard and fundamental management strategies for diabetic foot encompass debridement, revascularization, systemic antibiotic therapy, and stringent glycemic control. These interventions, while essential, are costly and require frequent hospitalizations (8). While existing treatments featuring tissue repair or the use of anti-inflammatory agents can be beneficial in closing or managing the progression of diabetic foot, most of these interventions are not well supported by clinical evidence (9). Furthermore, reports indicate that ulcer recovery with these therapies is inefficient and takes a long time (10). The high occurrence of lower limb ulcers and amputations in people with diabetes highlights an urgent need for improved treatments.

The standard strategy for preventing diabetes-related complications, as recommended by international guidelines, involves the application of glucose-lowering treatments to attain optimal glycemic levels and the reduction of modifiable risk factors (11). From a historical perspective, the glucose-lowering agents most frequently prescribed are metformin, insulin, and sulfonylureas (SU). Despite the proven efficacy of these drugs in reducing the risk of diabetic complications, their use is not without significant side effects, including hypoglycemia, particularly with insulin, and weight gain with SU (12, 13).

In recent years, the therapeutic landscape for T2DM and its associated complications has undergone a dramatic transformation due to the influx of novel oral glucose-lowering agents (14). These medications, include dipeptidyl peptidase-4 inhibitors (DPP-4is), glucagon-like peptide-1 receptor agonists (GLP-1RAs), sodium-glucose cotransporter-2 inhibitors (SGLT2is), with some of these drugs showing not only antidiabetic properties but also potential benefits for end organs.

An increasing body of evidence supports the potential cardioprotective and renoprotective properties of novel glucose-lowering agents beyond glycemic control. Nonetheless, research into their protective effects against diabetic foot complications is scant, and there is a lack of systematic summary. In this paper, we present a comprehensive narrative review of the existing evidence on the protective effects of novel hypoglycemic agents—DPP-4is, GLP-1RAs, and SGLT2is—on diabetic foot, summarizing proposed mechanisms and clinical findings.

## 2 DPP-4is and diabetic foot

DPP-4is, known as conventional anti-hyperglycemic drugs, are globally utilized and recommended as the first-line therapy for T2DM patients by the American Association of Clinical Endocrinologists (15). DPP-4is are progressively gaining prominence in the management of T2DM, progressively supplanting sulfonylureas in many countries (16). This trend is attributable to the characteristics of DPP-4is, which include no weight gain or hypoglycemia, a favorable safety profile, and ease of use (17).

The pervasive expression of DPP4 suggests additional roles for this enzyme beyond the regulation of endogenous glucose levels. Beyond glucose-lowering properties, multiple studies have pointed out that DPP-4is may exert additional effects on diabetic foot protection. DPP4, expressed on endothelial and epithelial cells, lymphocytes, and fibroblasts, exerts a range of diverse effects (18). Research on both diabetic humans and mice suggests that DPP-4is may mitigate several risk factors associated with diabetic foot complications. Beyond their favorable impact on glucose regulation, DPP-4is have demonstrated a spectrum of effects on blood pressure, postprandial lipemia, body weight, inflammatory markers, endothelial function, and oxidative stress, ranging from neutral to modestly beneficial in patients with T2DM (19, 20). Even though each effect might seem modest in isolation, the hypothesis that their cumulative impact could yield positive outcomes for diabetic foot care is plausible. Recent studies conducted recently have shown a different pattern of DPP4 expression in wounds between diabetic and healthy mice (21). Furthermore, DPP4-knockout mice exhibited expedited wound healing (22), suggesting that DPP4 impedes wound repair and regeneration. Consequently, DPP-4is may offer therapeutic potential in promoting the healing process of diabetic foot (22).

### 2.1 Potential mechanisms of DPP-4is in diabetic foot healing or development

#### 2.1.1 Inducing keratinocyte epithelialmesenchymal transition (EMT)

The EMT refers to the complete process of phenotypic transformation in quiescent epithelial cells, playing a significant role in the healing of skin wounds. During this process, dormant keratinocytes migrate across the wound bed through EMT, thereby restoring the epidermal barrier integrity. Experimental studies demonstrate that saxagliptin administration in both animal models (diabetic mice with dorsal skin ulcers) and clinical trials (diabetic foot patients) modulates EMT-related protein expression, including a decrease in E-cadherin and an increase in vimentin. Concurrently, saxagliptin enhances stromal cell-derived factor-1α (SDF-1α) production in fibroblasts, which directly or indirectly induces keratinocyte EMT, thereby accelerating wound epithelialization (23). In obese diabetic mice, the use of the DPP4 inhibitor linagliptin also facilitates wound healing by boosting epithelialization and the development of myofibroblasts (21).

#### 2.1.2 Promoting endothelial progenitor cells (EPCs) mobilization

EPCs constitute a heterogeneous cell population derived from bone marrow, exerting vasculoprotective effects through promoting endothelial repair and neovascularization. EPC mobilization is mediated via the SDF-1α/chemokine receptor type 4 axis, which is modulated by DPP-4 enzymatic activity. Notably, studies reveal that DPP-4is modulate EPC dynamics and SDF-1α levels through mechanisms independent of HbA1c, indicating SDF-1α serves as the predominant regulator of EPC mobilization. This glucose-independent pathway exerts more critical effects on augmenting peripheral blood EPC counts compared to systemic glycemic control. As all DPP-4is (sitagliptin, vildagliptin, saxagliptin, alogliptin, and linagliptin) consistently elevate circulating EPCs and SDF-1α (24–28), these findings suggest this mechanism may represent a class-wide glycemic-independent pleiotropic effect, fostering a pro-healing microenvironment in diabetic wounds.

#### 2.1.3 Stimulating angiogenesis

In chronic diabetic skin ulcers, local tissue hypoxia initiates adaptive responses through HIF-1α activation. HIF-1α orchestrates VEGF induction and upregulates iNOS, while critically mediating EPCs recruitment. This transcription factor concurrently mitigates hypoxic injury in wound beds and potentiates angiogenesis. Experimental evidence indicates that vildagliptin therapy attenuates oxidative stress, thereby suppressing 20S proteasome activity. This proteolytic inhibition reduces HIF-1α degradation, amplifying VEGF expression, which drives neovascularization in ulcerated tissues, ultimately accelerating wound closure through enhanced capillary network formation (29). Experimental studies indicate that the level of HMGB1 in the skin wounds of diabetic mice decreases. Administering HMGB1 locally can increase vascular density and accelerate wound healing in the skin of diabetic mice (29). DPP-4 inhibits HMGB1-induced endothelial cell migration and angiogenesis. However, after treatment with DPP-4is, serum levels of intact HMGB1 increase, reversing the inhibitory effect of DPP-4 on HMGB1-induced angiogenesis, thereby promoting wound neovascularization (30).

#### 2.1.4 Suppressing matrix metalloproteinase (MMP) expression in wound tissue

MMPs and tissue inhibitors of MMPs (TIMPs) are essential enzymes for wound healing, and their levels are inversely related. A disruption in the balance between MMPs and TIMPs can interfere with the cellular scaffolding needed for wound healing, resulting in impaired recovery (31). Studies have found that alogliptin can promote wound healing by inhibiting lipopolysaccharide-mediated extracellular signal-regulated kinase phosphorylation, suppressing macrophage DNA synthesis, and rebalancing the levels of MMPs and TIMPs (32).

Summaries of the potential mechanisms of DPP-4is in diabetic foot healing or development are shown in **Table 1**.

**Table 1:** Beneficial effects on outcomes in diabetic foot.

Classification	Compound	Effect	Reference
DPP-4is	Saxagliptin	Enhance SDF-1α production in fibroblasts, Induces keratinocyte EMT and migration	Long M, Cai L et al., 2017 (23)
		Increase FMD and EPCs number	Li F, Chen J et al., 2017 (28)
	Sitagliptin	Elevate circulating EPCs and SDF-1α	Fadini GP, Boscaro E et al., 2010 (24)
		Improve keratinocyte migration, Induce keratinocyte EMT, Induce SDF-1α expression	Long M, Cai L et al., 2017 (23)
	Linagliptin	Promote epithelialization and the formation of myofibroblasts, Attenuate inflammatory response, Induce GLP-1 expression	Schürmann C, Linke A et al., 2012 (21)
		Increase CD34(+)CD133(+) progenitor cells, CD34(+)KDR(+) EPCs, and CX3CR1 monocytes.	Fadini GP, Bonora BM et al., 2016 (27)
		Improve keratinocyte migration	Long M, Cai L et al., 2017 (23)
	Alogliptin	Suppress TLR4-mediated ERK activation and ERK-dependent MMP expression	Ta NN, Li Y et al., 2010 (32)
		Improve keratinocyte migration	Long M, Cai L et al., 2017 (23)
	Vidagliptin	Improve keratinocyte migration	Long M, Cai L et al., 2017 (23)
GLP-1RAs	Exendin-4	Induce quantitative increase in fibroblasts/myofibroblasts and vessel density	Bacci S, Laurino A et al., 2015 (38)
		Improve migration, invasion and proliferation of human endothelial cells	Seo E, Lim JS et al., 2017 (46)
		Attenuate inflammatory response and enhance angiogenesis during the early proliferation phase	Roan JN, Cheng HN et al., 2017 (44)
		Enhance transforming growth factor-β/matrix metalloproteinase-mediated regeneration during the maturation phase	Roan JN, Cheng HN et al., 2017 (44)
	Liraglutide	AMPK-dependent endothelial protection and pro-angiogenic effects	Huang H, Wang L et al., 2021 (48)
		Stimulate keratinocyte migration through the activation of the PI3K/Akt pathway	Nagae K, Uchi H et al., 2018 (50)
		Stimulate the invasion, migration, and proliferation of HUVECs, Decrease the level of miR-29b-3p through the AKT/GSK-3β/β-catenin pathway to regulate the biological function of ECs	Yu M, Huang J et al., 2020 (47)
SGLT2is	Empagliflozin	Improve sciatic nerve histopathological alterations, scoring, myelination, nerve fiber count, and nerve conduction velocity, Alleviate responses to different nociceptive stimuli along with improved motor coordination	Abdelkader NF, Elbaset MA et al., 2022 (66)

DPP-4is, dipeptidyl peptidase-4 inhibitors; GLP-1RAs, glucagon-like peptide-1 receptor agonists; SGLT2is, sodium-glucose cotransporter-2 inhibitors; SDF-1α, stromal cell-derived factor-1α; EMT, epithelial-mesenchymal transition; EPCs, endothelial progenitor cells; ECs, endothelial cells; MMP, matrix metalloproteinase; FMD, flow-mediated dilation.

### 2.2 Clinical evidence of DPP-4is in diabetic foot healing or development

Scholars have utilized it for the management of refractory diabetic skin ulcers, yielding significant clinical effectiveness. Saxagliptin demonstrates significant potential in improving the healing rate of diabetic foot ulcers (DFUs) and reducing the healing duration (23). The vildagliptin group had notably higher granulation scores and complete wound healing rates compared to the control group. Moreover, there was a significant reduction in the occurrence of adverse events related to ulcers, such as ulcerative wound infections, cellulitis, and osteomyelitis (29). Additionally, the use of DPP-4is was associated with a lower risk of DFU and DFU-related outcomes, including lower limb amputation (LLA), DFU-related hospitalizations, and mortality, in comparison to sulfonylureas (33, 34). Moreover, a study revealed that users of DPP-4is exhibited a reduced risk of developing peripheral arterial disease and LLA compared to nonusers (35). Clinical applications have unveiled no severe adverse events, implying DPP-4is may serve as a novel adjunct therapeutic strategy for managing DFU in clinical settings. **Table 2** presents a range of clinical evidence on the use of DPP-4is in the healing and development of diabetic foot.

**Table 2:** Clinical outcomes of novel glucose-lowering agents in diabetic foot management.

Intervention	Comparison	Result	Conclusion	Reference
DPP-4is	sulfonylureas	HR, 0.88; 95% CI, 0.79, 0.97 HR, 0.83; 95% CI, 0.67, 1.03 HR, 0.85; 95% CI, 0.73, 0.99 HR, 0.79; 95% CI, 0.75, 0.83	DFU↓ LLA^#^ Hospitalization↓ Mortality↓	Werkman, Nikki C C et al., 2024 (33)
	placebo	HR, 0.65; 95% CI, 0.54, 0.79	LLA↓	Chang, Chun-Chin et al., 2017 (35)
	placebo	Wound closure rate: 31% in the vildagliptin group, 15% in the control group (week 12)	Ulcer-related adverse events (local wound infection, osteomyelitis, and cellulitis) ↓	Marfella, Raffaele et al., 2012 (29)
	placebo	Rate of wound healing↑		Long, Min et al., 2018 (23)
GLP-1RAs	sulfonylureas	HR,0.57; 95% CI, 0.39, 0.84	LLA↓	Werkman, Nikki C C et al., 2023 (53)
	insulin	HR, 0.44; 95% CI, 0.32, 0.60 HR, 0.46; 95% CI, 0.33, 0.65 HR, 0.60; 95% CI, 0.46, 0.77 HR, 0.53; 95% CI, 0.46, 0.62	DFU↓ LLA↓ Hospitalization↓ Mortality↓	Werkman, Nikki C C et al., 2024 (33)
	placebo	HR, 0.65; 95% CI, 0.45, 0.95	Amputation↓	Dhatariya, Ketan et al., 2018 (51)
	placebo	HR, 0.50; 95% CI, 0.54, 0.74	Amputation↓	Schäfer, Zeinab et al., 2023 (52)
	DPP-4i	HR, 0.63; 95% CI, 0.41, 0.96	MALE↓	Lin, Donna Shu-Han et al., 2021 (54)
	SGLT2i	HR, 0.80; 95% CI, 0.67, 0.96	MALE↓	Lin, Donna Shu-Han et al., 2022 (55)
SGLT2is	sulfonylureas	HR, 1.10; 95% CI, 0.71, 1.70	LLA^#^	Werkman, Nikki C C et al., 2023 (53)
	sulfonylureas	HR, 0.70; 95% CI, 0.38, 1.29	LLA^#^	Werkman, Nikki C C et al., 2021 (86)
	sulfonylureas	HR, 0.74; 95% CI, 0.57, 0.96	LEA↓	Dawwas, Ghadeer K et al., 2019 (87)
	GLP-1RA	HR, 1.27; 95% CI, 0.63, 2.55	LEA^#^	Lee, Yen-Chieh et al., 2022 (80)
	GLP-1RA	HR, 1.47; 95% CI, 0.64, 3.36	Amputation^#^	Chang, Hsien-Yen et al., 2018 (79)
	GLP-1RA	HR, 2.32; 95% CI, 1.37, 3.91	LLA↑	Ueda, Peter et al., 2018 (83)
	GLP-1RA	HR, 1.73; 95% CI, 1.30, 2.29 (age >65 with baseline cardiovascular disease)	Amputation↑	Fralick, Michael et al., 2020 (82)
	GLP-1RA	RD, 0.90; 95% CI, 0.10, 1.70 (Age ≥66)	LLA↑	Patorno, Elisabetta et al., 2022 (89)
	GLP-1RA	HR, 1.65; 95% CI, 1.22, 2.23	LLA↑	Fu, Edouard L et al., 2023 (85)
	DPP-4i	HR, 0.80; 95% CI, 0.42, 1.53	LEA^#^	Lee, Yen-Chieh et al., 2022 (80)
	DPP-4i	HR, 1.50; 95% CI, 0.85, 2.67	Amputation^#^	Chang, Hsien-Yen et al., 2018 (79)

DPP-4is, dipeptidyl peptidase-4 inhibitors; GLP-1RAs, glucagon-like peptide-1 receptor agonists; SGLT2is, sodium-glucose cotransporter-2 inhibitors; MALE, defined as the composite of newly diagnosed critical limb ischaemia, percutaneous transluminal angioplasty or peripheral bypass for peripheral artery disease, and nontraumatic amputation; LEA, lower extremity amputation; LLA, lower limb amputation; ^#^, not associated.

↑ indicates that compared to using Comparison, using Intervention is associated with a higher risk of a certain outcome. ↓ indicates that compared to using Comparison, using Intervention is associated with a lower risk of a certain outcome.

DPP-4is have demonstrated promising outcomes by targeting various factors involved in wound healing in animal research, with clinical evidence indicating favorable outcomes in diabetic wound management. In basic research, comprehending the bioactive substrate of the DPP-4 enzyme, which plays an integral role in wound healing, represents a critical research question that demands elucidation. DPP-4 is complex in its action because of its enzymatic properties and its role in signaling within cells. Further studies exploring DPP-4’s role in wound healing, by examining potential substrates, its influence on immune function, and possible off-target effects, will be instrumental in comprehending the role of DPP-4is in the treatment of diabetic foot. Furthermore, the clinical data pertaining to the use of DPP-4is do not rule out the possibility that the glucose-lowering effects of incretin-based treatments aid in improved wound healing. A comprehensive understanding of this question necessitates further experimentation with the localized, topical application of DPP-4is directly to wound tissues. The topical delivery of drugs is a promising method that may reduce the incidence of the agent’s adverse effects. Wound care is a crucial aspect of managing DFU. Combining incretin-based therapy with known topical treatments may offer an innovative approach to enhance the management of DFU. In addition, there is a currently shortage of multicenter randomized controlled trials with large sample sizes and long follow-up periods to further confirm the therapeutic efficacy and safety of DPP-4is. We suggest conducting extensive clinical trials with a range of clinically used DPP-4is medications to confirm their effectiveness in treating diabetic foot. We are confident that such trials can benefit the development of clinical individualized treatments in future settings.

## 3 GLP-1RAs and diabetic foot

GLP-1RAs rank among the most promising hypoglycemic drugs for treating T2DM and obesity. Together with DPP-4is, these novel classes of glucose-lowering drugs exert their effects through the incretin pathway. GLP-1RAs and DPP-4is are highly recommended as adjunctive therapies to metformin due to their several benefits over alternative diabetes treatments. These agents are especially attractive as additional therapies because they boost insulin release based on glucose levels, reducing the risk of hypoglycemia. GLP-1RAs seem to surpass DPP-4is in decreasing hemoglobin A1c levels and offer cardiovascular benefits (36). Furthermore, administering a GLP-1RA injection once a week is more convenient and enhances patient compliance (37).

An increasing amount of evidence suggests that the advantages of GLP-1 and GLP-1RAs extend beyond just weight loss and glycemic control. GLP-1 receptors are identified in keratinocytes, fibroblasts, immune cells, and endothelial cells situated in the papillary and reticular areas of the dermis, which indicates a possible direct advantage for wound healing by activating GLP-1 receptors. The activation of GLP-1 receptors has been found to promote wound healing in mice with normal glucose levels (38). Additionally, GLP-1RAs have been reported to exert antioxidant (39, 40), anti-inflammatory (41), and proangiogenic effects (42, 43), indicating a possible therapeutic benefit for healing wounds in diabetic conditions.

### 3.1 Potential mechanisms of GLP-1RAs in diabetic foot healing or development

#### 3.1.1 Exerting anti-inflammatory and antioxidant effects

The development of diabetic foot is driven by the complex interactions among hyperglycemia, inflammation, and oxidative stress. Concurrent oxidative stress and hyperinflammation typically manifest in the tissues affected by diabetic foot, further exacerbating wound deterioration. Administering exendin-4 successfully promoted wound healing in diabetic rats. It notably reduced cytokine production and limited the infiltration of inflammatory cells at the wound sites, indicating its anti-inflammatory and protective benefits during the healing process (38, 44). In addition to inhibiting the inflammatory response, exendin-4 has been reported to enhance the recovery of chronic wounds by improving antioxidant capacity. Exendin-4 intervention reduced the expression of heme oxygenase-1, an enzyme involved in oxidative stress (45), thereby exerting an antioxidant effect on excisional wound tissue (44).

#### 3.1.2 Promoting angiogenesis

Angiogenesis is vital for wound healing and occurs due to the interaction between endothelial cells and growth factors. A reduction in angiogenic capacity is a primary factor contributing to the development of chronic wounds in diabetes. *In vitro* studies examining the influence of GLP-1RAs on angiogenesis found that treatment with exendin-4 increased the count of lumenized vessels. Additionally, it enhanced the invasion, migration, and proliferation of HUVECs (46, 47). Moreover, exendin-4 facilitated the mobilization of circulating EPCs, identified by CD34+/KDR+ markers, in skin wounds. This activity promoted angiogenesis and sped up the wound healing process (44). In db/db mice, the administration of liraglutide alleviated endothelial dysfunction caused by hyperglycemia and increased angiogenesis in diabetic wounds. This improvement occurred via the Hypoxia-Inducible Factor/Heme Oxygenase-1 pathway (48). Additionally, liraglutide effectively reduced the levels of miRNA-29b-3p in rats induced with STZ. This reduction led to the regulation of endothelial cell functions by targeting the Akt/Glycogen Synthase Kinase-3β/β-catenin pathway (47).

#### 3.1.3 Enhancing re-epithelialization

Wound closure entails coordinated execution of multiple physiological mechanisms, including wound contraction, keratinocyte migration, granulation tissue formation, and the remodeling of extracellular matrix (ECM) proteins. TGF-β is crucial for wound healing as it boosts MMP activity, whereas decreased TGF-β activity is associated with poor wound healing. The administration of Exendin-4 exhibited regulatory effects on TGF-β and MMP expression dynamics during the crucial remodeling phase of diabetic wounds, thereby facilitating ECM remodeling and accelerating the healing of diabetic wounds (44). Additionally, exendin-4 administration enhanced the chemotactic migration and recruitment of dermal fibroblasts/myofibroblasts, thereby improving wound repair in diabetic rats (49). Research has shown that liraglutide can stimulate keratinocyte migration through the activation of the PI3K/Akt pathway, highlighting the potential of GLP-1RAs to enhance epithelial regeneration in diabetic wounds (50).

Summaries of the potential mechanisms of GLP-1RAs in diabetic foot healing or development are shown in **Table 1**.

### 3.2 Clinical evidence of GLP-1RAs in diabetic foot healing or development

The clinical outcomes of GLP-1RAs on DFU have yet to be thoroughly investigated. GLP-1RAs have undergone more extensive investigation concerning their association with the risk of DFU-related outcomes (LLA). A subsequent analysis of the LEADER trial showed that liraglutide reduced the risk of LLA compared to a placebo (51). Other studies have shown a decreased risk of LLA with GLP-1RAs compared to patients without treatment (52), SU use (53), DPP-4is use (54), and SGLT2is use (55). In the latest research, we found that GLP-1RAs were linked to a decreased risk of DFU, LLA, DFU-related hospitalization, and mortality associated with the use of GLP-1RAs compared to insulin therapy (33). **Table 2** presents a range of clinical evidence on the use of GLP-1RAs in the healing and development of diabetic foot.

Considering the above, large-scale clinical trials are essential to validate the protective effects of GLP-1RAs on DFU and its related outcomes. Additionally, research into the use of GLP-1-based therapies, applied topically, for patients with DFU carries substantial significance. Once more proof is collected to validate these beneficial impacts, it could introduce new auxiliary methods to enhance the treatment efficacy of resistant diabetic foot.

## 4 SGLT2is and diabetic foot

SGLT2is represent a novel class of pharmacological agents for the management of T2DM. These drugs produce a hypoglycemic effect by inhibiting the reabsorption of glucose and sodium in the proximal renal tubules, independently of insulin action (56). Consequently, they facilitate weight loss without triggering hypoglycemia. Moreover, by diminishing glucotoxicity, they indirectly enhance β-cell function and insulin sensitivity (57, 58). SGLT2is are applicable across all stages of T2DM’s natural history, with the exception of cases involving moderate to severe chronic kidney disease (59, 60). Overall, this pharmacological class offers a favorable efficacy-to-risk ratio (61).

Diabetic foot development and progression are driven by interconnected factors, including inflammatory changes, neuropathy, arteriopathy, and metabolic causes. SGLT2is have demonstrated an ability to decelerate the progression of atherosclerosis by lowering oxidative stress and inflammation, independent of glucose control (62). These medications improve endothelial function, decrease oxidative damage, lower levels of proinflammatory cytokines, and block major inflammatory pathways. In addition, they enhance mitochondrial performance, promote autophagy, and help stabilize atherosclerotic plaques, offering potential cardiovascular protective effects for individuals with diabetes. Recent research suggests that SGLT2is could provide therapeutic advantages for diabetic neuropathy by targeting inflammation and oxidative stress mechanisms, apart from their role in glucose management. Furthermore, SGLT2is demonstrate potential in addressing various complications of diabetes, including improving peripheral artery disease (PAD) outcomes and promoting wound healing. Current evidence supports their neuroprotective, immunomodulatory, and vascular benefits, suggesting a potential therapeutic advantage for diabetic foot management (63).

### 4.1 SGLT2is and neuropathy

Neuropathy is the primary precipitating factor in diabetic foot development (2). Prolonged hyperglycemia damages peripheral nerves, resulting in diminished or absent sensation of pain and temperature in the feet (64). Consequently, patients are unable to perceive minor injuries, which can easily progress to ulcers (65). The current body of literature features a limited number of studies on how SGLT2is affect diabetic neuropathy at the molecular level. Recent research investigated the ability of empagliflozin to ameliorate streptozotocin-induced diabetic peripheral neuropathy (DPN) in rats, elucidating its signaling mechanisms in detail. The findings indicated that empagliflozin had a protective role against DPN, independent of its effects on lowering blood glucose levels, likely by affecting the AMPK pathway to influence inflammatory, oxidative stress, autophagy, and extracellular matrix remodeling (66). Additionally, in a study conducted over three years, SGLT2is demonstrated a notable improvement in several neuropathy outcome measures. This was reflected in the mean Z-score derived from eight neurophysiological assessments: sural sensory nerve conduction velocity and amplitude, median motor nerve conduction velocity and amplitude, R-R interval variation coefficient, vibration detection threshold, and thresholds for cold and warm perception (67). A meta-analysis evaluating the benefits of using SGLT2is for managing diabetic neuropathy revealed that SGLT2is may offer neuroprotective effects by significantly increasing sensory and motor nerve conduction velocity, reducing sympathetic nervous system activity, and alleviating the clinical symptoms of DPN (68).

### 4.2 SGLT2is and immunomodulation

Macrophages, fibroblasts, regulatory T cells, and other repair cells work together to promote efficient healing and form a healthy skin barrier following an injury. Immune dysregulation during wound healing can lead to chronic wounds. In diabetic patients, hyperglycemia influences wound pathophysiology by interfering with the immune system and leading to nerve and blood flow issues, which complicate healing (69). Few studies have explored the role of SGLT2 in modulating the immune system. The immunomodulatory effects of SGLT2is are primarily seen in their interaction with macrophages and T lymphocytes. Empagliflozin has been demonstrated to lower M1 macrophage expression and promote M2 macrophage polarization, offering protective effects against inflammation in liver macrophages and white adipose tissue. This was demonstrated in a study involving mice made obese through a high-fat diet (70). Furthermore, Empagliflozin has been observed to boost regulatory T cell subsets while reducing proinflammatory Th1 and Th17 subsets. It achieves this by influencing the CD4+ response via the mTOR signaling pathway (71). Another study revealed that canagliflozin has the ability to decrease the production of inflammatory proteins by CD4+ cells in individuals suffering from autoimmune disorders. This suggests that such medications might contribute to the regulation of immune and autoimmune responses (72).

### 4.3 SGLT2is and risk of amputation

The link between SGLT2i use and the occurrence of LLA continues to be ambiguous. The preliminary findings of an elevated risk of LLA linked to canagliflozin, an SGLT2 inhibitor, reported in the Canagliflozin Cardiovascular Assessment Study Program (73), generated significant concern. Later research similarly noted a modest rise in amputation risk among patients taking SGLT2is. This increase was mainly attributed to the use of canagliflozin (74, 75). In response, a series of studies emerged to rigorously explore this potential association. While some studies have reported conflicting outcomes, meta-analyses have demonstrated that there is no heightened risk of LLA when using SGLT2is as opposed to other glucose-lowering medications or a placebo (76–78). Several studies have explored the association between SGLT2is and the risk of LLA or lower extremity minor and major amputation (LEA), using GLP-1RAs as the control group. Some studies show no significant difference in risk (79–81), whereas others propose that SGLT2is might be linked to a higher risk (82–85). Comparative analysis of SGLT2is and SU in the context of LLA risk has been limited to a few cohort studies, with two indicating comparable risk and one suggesting a lower risk associated with SGLT2is (53, 86, 87). SGLT2is have been shown to be just as safe as DPP-4is and do not raise the risk of lower limb complications (88). Additionally, there were no observed differences in the safety of empagliflozin compared to DPP-4is regarding the incidence of LLA or fractures (89). A recent retrospective study found that SGLT2is may safely and effectively reduce the risk of amputation in patients with recent diabetic foot complications who have a low cardiovascular risk (90).The diversity of findings may stem from variations in study populations and methodologies, particularly due to the use of distinct reference groups. **Table 2** presents a range of clinical evidence on the use of SGLT2is in the healing and development of diabetic foot.

While the influence of SGLT2is on LLA remains a contentious issue, the principal mechanism suggested for this conjectured outcome is hypovolemia with reduced tissue perfusion, though this mechanism has yet to be substantiated. The use of SGLT2is has been associated with heightened glucose excretion, which may result in increased sodium excretion and osmotic diuresis. This sequence of events could potentially diminish peripheral tissue perfusion, leading to necrosis and, in severe cases, necessitating amputation (91). The mechanism is supported by the observed elevated risk of LLA in patients with T2DM who use diuretics compared to non-users. Additionally, there is a correlation between reductions in body weight and blood pressure and a decrease in lower limb complications in treated patients (74, 92).

The recent study found no increased risk of LLA associated with the use of SGLT2is, nor was there a correlation between current SGLT2is exposure and the incidence of LLA amidst signs of hypovolemia. Ultimately, the potential association between SGLT2is and LLA, as well as the potential role of hypovolemia, is still not well understood.

It is pivotal to underscore that there appear to be no investigations concerning SGLT2is in the context of diabetic wound healing within animal or cellular models. Further research is required to derive more definitive conclusions regarding the precise mechanisms and the impact of these agents on diabetic foot conditions. In conclusion, current evidence supports the individual neuroprotective, immunomodulatory, and vascular benefits of SGLT2is. Nonetheless, given the scarcity of clinical data, evaluating the potential effects of this drug class on diabetic foot prevention remains challenging. The potential but unverified risk of limb amputation associated with canagliflozin might have deterred researchers from conducting studies focused on diabetic foot. Considering this evidence, we recommend caution in the use of SGLT2is in patients with lower limb ischemic disease or diabetic foot. Current evidence regarding infection risks associated with SGLT2is use in diabetic patients has primarily centered on genitourinary tract infections, suggesting that only patients with certain susceptibility factors, such as those more prone to genital and urinary tract infections (e.g., poor glycemic control, neurogenic bladder, abnormalities in the urinary tract, frequent vaginal infections, and neurogenic bladder), may benefit from avoiding SGLT2is.

## 5 Conclusion

Contemporary basic and clinical research reveals that DPP-4is and GLP-1RAs have demonstrated promising results in the management of diabetic foot, despite existing controversies regarding SGLT2is. Their action on multiple target enzymes involved in the chronic inflammation associated with diabetic foot could potentially facilitate wound healing. While Effective regulation of blood glucose inherently aids in the healing of wounds for diabetic patients, considering the current challenges of foot ulcers, their enduring nature, and the high frequency of amputations in this population, alongside ineffective diabetic foot management strategies, it is imperative to address wound healing characteristics of currently employed hypoglycemic drugs. Further research in this field may forge the path for an effective therapeutic approach to the issue of diabetic foot (93). Therapies based on novel hypoglycemic drugs can offer significant therapeutic strategies to diabetic patients and those with an increased risk of diabetic foot, fostering the advancement of personalized treatment methodologies for individuals with diabetes.

## Group members of International Flow and Toe Research Team (iFORT)

Dongfeng Tang, Canlong Wang, Zhangrong Xu, Yufeng Jiang, Xiong Chen, Zhiqiang Huo, Yun Gao, Johnson Boey, Yan Chen, Anxin Li, Min Wang, Yi Yuan, Charareh Pourzand, Qiu Chen, Ya Liu, Rijian Song, Wenxin Wang, Zhongming Wu, Lin Li, Li Zhang, Mengchen Zou. School of Life Course and Population Health Sciences, King’s College London, London, UK (Dongfeng Tang, Canlong Wang), Diabetes Centre, The 306th Hospital of PLA, Beijing, China (Zhangrong Xu), Department of Tissue Regeneration and Wound Repair, The First Medical Center, Chinese PLA General Hospital, Beijing, China (Yufeng Jiang). Center of Wound Repair and Regeneration, the First Affiliated Hospital of Wenzhou Medical University, Wenzhou, Zhejiang, China (Xiong Chen), Queen Mary University of London, London, UK (Zhiqiang Huo), Department of Endocrinology and Metabolism, Diabetic Foot Care Center, West China Hospital, Sichuan University, Chengdu, Sichuan, China (Yun Gao), Department of Podiatry, National University Hospital Singapore, Singapore, Singapore (Johnson Boey), Diabetic Foot Medical Research Center, Department of Endocrinology, Chongqing University Central Hospital, Chongqing University, Chongqing, China (Yan Chen, Anxin Li, Shunli Rui, Min Wang, Yi Yuan), Department of Life Sciences, University of Bath, Bath, UK (Charareh Pourzand), Department of Endocrinology, Hospital of Chengdu University of Traditional Chinese Medicine, Chengdu, China (Qiu Chen, Ya Liu), Charles Institute of Dermatology, School of Medicine, University College Dublin, Dublin 4, Ireland (Rijian Song, Wenxin Wang), Department of Endocrinology, Shandong Provincial Hospital Affiliated to Shandong First Medical University, Shandong First Medical University, Jinan, Shandong, China (Zhongming Wu), Department of Endocrinology, Zhejiang University School of Medicine Sir Run Shaw Hospital, Hangzhou, China (Lin Li), Guang ‘anmen Hospital, China Academy of Chinese Medical Sciences, Beijing, China (Li Zhang), Department of Endocrinology and Metabolism, Nanfang Hospital, Southern Medical University, Guangzhou, China (Mengchen Zou).

## References

[1] Cho NH Shaw JE Karuranga S Huang Y da Rocha Fernandes JD Ohlrogge AW. IDF Diabetes Atlas: Global estimates of diabetes prevalence for 2017 and projections for 2045. Diabetes Res Clin Pract. (2018) 138:271–81. doi: 10.1016/j.diabres.2018.02.023 29496507

[2] McDermott K Fang M Boulton AJM Selvin E Hicks CW. Etiology, epidemiology, and disparities in the burden of diabetic foot ulcers. Diabetes Care. (2023) 46:209–21. doi: 10.2337/dci22-0043 PMC9797649 36548709

[3] Yang M Deng B Hao W Jiang X Chen Y Wang M. Platelet concentrates in diabetic foot ulcers: A comparative review of PRP, PRF, and CGF with case insights. Regener Ther. (2025) 28:625–32. doi: 10.1016/j.reth.2025.02.005 PMC11955794 40166040

[4] Armstrong DG Boulton AJM Bus SA. Diabetic foot ulcers and their recurrence. N Engl J Med. (2017) 376:2367–75. doi: 10.1056/NEJMra1615439 28614678

[5] Armstrong DG Swerdlow MA Armstrong AA Conte MS Padula WV Bus SA. Five year mortality and direct costs of care for people with diabetic foot complications are comparable to cancer. J Foot Ankle Res. (2020) 13:16. doi: 10.1186/s13047-020-00383-2 32209136 PMC7092527

[6] Tola A Regassa LD Ayele Y. Prevalence and associated factors of diabetic foot ulcers among type 2 diabetic patients attending chronic follow-up clinics at governmental hospitals of Harari Region, Eastern Ethiopia: A 5-year (2013-2017) retrospective study. SAGE Open Med. (2021) 9:2050312120987385. doi: 10.1177/2050312120987385 33552513 PMC7838876

[7] Rui S Dai L Zhang X He M Xu F Wu W. Exosomal miRNA-26b-5p from PRP suppresses NETs by targeting MMP-8 to promote diabetic wound healing. J Control Release. (2024) 372:221–33. doi: 10.1016/j.jconrel.2024.06.050 38909697

[8] Yazdanpanah L Nasiri M Adarvishi S. Literature review on the management of diabetic foot ulcer. World J Diabetes. (2015) 6:37–53. doi: 10.4239/wjd.v6.i1.37 25685277 PMC4317316

[9] Chen T Song P He M Rui S Duan X Ma Y. Sphingosine-1-phosphate derived from PRP-Exos promotes angiogenesis in diabetic wound healing via the S1PR1/AKT/FN1 signalling pathway. Burns Trauma. (2023) 11:tkad003. doi: 10.1093/burnst/tkad003 37251708 PMC10208895

[10] Monami M Ragghianti B Nreu B Lorenzoni V Pozzan M Silverii A. Major amputation in non-healing ulcers: outcomes and economic issues. Data from a cohort of patients with diabetic foot ulcers. Int J Low Extrem Wounds. (2025) 24:235–44. doi: 10.1177/15347346221097283 35477285

[11] Templer S Abdo S Wong T. Preventing diabetes complications. Intern Med J. (2024) 54:1264–74. doi: 10.1111/imj.16455 39023283

[12] Frier BM. Hypoglycaemia in diabetes mellitus: epidemiology and clinical implications. Nat Rev Endocrinol. (2014) 10:711–22. doi: 10.1038/nrendo.2014.170 25287289

[13] Bennett WL Maruthur NM Singh S Segal JB Wilson LM Chatterjee R. Comparative effectiveness and safety of medications for type 2 diabetes: an update including new drugs and 2-drug combinations. Ann Intern Med. (2011) 154:602–13. doi: 10.7326/0003-4819-154-9-201105030-00336 PMC3733115 21403054

[14] Kahn SE Cooper ME Del Prato S. Pathophysiology and treatment of type 2 diabetes: perspectives on the past, present, and future. Lancet. (2014) 383:1068–83. doi: 10.1016/s0140-6736(13)62154-6 PMC4226760 24315620

[15] Garber AJ Abrahamson MJ Barzilay JI Blonde L Bloomgarden ZT Bush MA. American Association of Clinical Endocrinologists’ comprehensive diabetes management algorithm 2013 consensus statement–executive summary. Endocr Pract. (2013) 19:536–57. doi: 10.4158/ep13176.Cs PMC4142590 23816937

[16] Garber AJ Abrahamson MJ Barzilay JI Blonde L Bloomgarden ZT Bush MA. Consensus statement by the american association of clinical endocrinologists and american college of endocrinology on the comprehensive type 2 diabetes management algorithm - 2018 executive summary. Endocr Pract. (2018) 24:91–120. doi: 10.4158/cs-2017-0153 29368965

[17] Scheen AJ. Safety of dipeptidyl peptidase-4 inhibitors for treating type 2 diabetes. Expert Opin Drug Saf. (2015) 14:505–24. doi: 10.1517/14740338.2015.1006625 25630605

[18] Mentzel S Dijkman HB Van Son JP Koene RA Assmann KJ. Organ distribution of aminopeptidase A and dipeptidyl peptidase IV in normal mice. J Histochem Cytochem. (1996) 44:445–61. doi: 10.1177/44.5.8627002 8627002

[19] Scheen AJ. A review of gliptins for 2014. Expert Opin Pharmacother. (2015) 16:43–62. doi: 10.1517/14656566.2015.978289 25381751

[20] Makdissi A Ghanim H Vora M Green K Abuaysheh S Chaudhuri A. Sitagliptin exerts an antinflammatory action. J Clin Endocrinol Metab. (2012) 97:3333–41. doi: 10.1210/jc.2012-1544 PMC3431580 22745245

[21] Schürmann C Linke A Engelmann-Pilger K Steinmetz C Mark M Pfeilschifter J. The dipeptidyl peptidase-4 inhibitor linagliptin attenuates inflammation and accelerates epithelialization in wounds of diabetic ob/ob mice. J Pharmacol Exp Ther. (2012) 342:71–80. doi: 10.1124/jpet.111.191098 22493041

[22] Baticic Pucar L Pernjak Pugel E Detel D Varljen J. Involvement of DPP IV/CD26 in cutaneous wound healing process in mice. Wound Repair Regener. (2017) 25:25–40. doi: 10.1111/wrr.12498 27868279

[23] Long M Cai L Li W Zhang L Guo S Zhang R. DPP-4 inhibitors improve diabetic wound healing via direct and indirect promotion of epithelial-mesenchymal transition and reduction of scarring. Diabetes. (2018) 67:518–31. doi: 10.2337/db17-0934 29254987

[24] Fadini GP Boscaro E Albiero M Menegazzo L Frison V de Kreutzenberg S. The oral dipeptidyl peptidase-4 inhibitor sitagliptin increases circulating endothelial progenitor cells in patients with type 2 diabetes: possible role of stromal-derived factor-1alpha. Diabetes Care. (2010) 33:1607–9. doi: 10.2337/dc10-0187 PMC2890368 20357375

[25] Negro R Greco EL Greco G. Active stromal cell-derived factor 1α and endothelial progenitor cells are equally increased by alogliptin in good and poor diabetes control. Clin Med Insights Endocrinol Diabetes. (2017) 10:1179551417743980. doi: 10.1177/1179551417743980 29225483 PMC5714079

[26] Dei Cas A Spigoni V Cito M Aldigeri R Ridolfi V Marchesi E. Vildagliptin, but not glibenclamide, increases circulating endothelial progenitor cell number: a 12-month randomized controlled trial in patients with type 2 diabetes. Cardiovasc Diabetol. (2017) 16:27. doi: 10.1186/s12933-017-0503-0 28231835 PMC5324295

[27] Fadini GP Bonora BM Cappellari R Menegazzo L Vedovato M Iori E. Acute effects of linagliptin on progenitor cells, monocyte phenotypes, and soluble mediators in type 2 diabetes. J Clin Endocrinol Metab. (2016) 101:748–56. doi: 10.1210/jc.2015-3716 26695864

[28] Li F Chen J Leng F Lu Z Ling Y. Effect of saxagliptin on circulating endothelial progenitor cells and endothelial function in newly diagnosed type 2 diabetic patients. Exp Clin Endocrinol Diabetes. (2017) 125:400–7. doi: 10.1055/s-0042-124421 28407661

[29] Marfella R Sasso FC Rizzo MR Paolisso P Barbieri M Padovano V. Dipeptidyl peptidase 4 inhibition may facilitate healing of chronic foot ulcers in patients with type 2 diabetes. Exp Diabetes Res. (2012) 2012:892706. doi: 10.1155/2012/892706 23197976 PMC3503302

[30] Marchetti C Di Carlo A Facchiano F Senatore C De Cristofaro R Luzi A. High mobility group box 1 is a novel substrate of dipeptidyl peptidase-IV. Diabetologia. (2012) 55:236–44. doi: 10.1007/s00125-011-2213-6 21656024

[31] Li Z Guo S Yao F Zhang Y Li T. Increased ratio of serum matrix metalloproteinase-9 against TIMP-1 predicts poor wound healing in diabetic foot ulcers. J Diabetes Complications. (2013) 27:380–2. doi: 10.1016/j.jdiacomp.2012.12.007 23357650

[32] Ta NN Li Y Schuyler CA Lopes-Virella MF Huang Y. DPP-4 (CD26) inhibitor alogliptin inhibits TLR4-mediated ERK activation and ERK-dependent MMP-1 expression by U937 histiocytes. Atherosclerosis. (2010) 213:429–35. doi: 10.1016/j.atherosclerosis.2010.08.064 20843518

[33] Werkman NCC Driessen JHM Klungel OH Schaper NS Souverein PC Stehouwer CDA. Incretin-based therapy and the risk of diabetic foot ulcers and related events. Diabetes Obes Metab. (2024) 26:3764–80. doi: 10.1111/dom.15721 38951877

[34] Yu WH Zhang T Xu H. Role of dipeptidyl dipeptidase 4 inhibitors in the management of diabetic foot. Int J Low Extrem Wounds. (2022) 23(4):577–84. doi: 10.1177/15347346221082776 35225718

[35] Chang CC Chen YT Hsu CY Su YW Chiu CC Leu HB. Dipeptidyl peptidase-4 inhibitors, peripheral arterial disease, and lower extremity amputation risk in diabetic patients. Am J Med. (2017) 130:348–55. doi: 10.1016/j.amjmed.2016.10.016 27884648

[36] Prasad-Reddy L Isaacs D. A clinical review of GLP-1 receptor agonists: efficacy and safety in diabetes and beyond. Drugs Context. (2015) 4:212283. doi: 10.7573/dic.212283 26213556 PMC4509428

[37] Polonsky WH Fisher L Hessler D Bruhn D Best JH. Patient perspectives on once-weekly medications for diabetes. Diabetes Obes Metab. (2011) 13:144–9. doi: 10.1111/j.1463-1326.2010.01327.x 21199266

[38] Bacci S Laurino A Manni ME Landucci E Musilli C De Siena G. The pro-healing effect of exendin-4 on wounds produced by abrasion in normoglycemic mice. Eur J Pharmacol. (2015) 764:346–52. doi: 10.1016/j.ejphar.2015.06.056 26164788

[39] Bułdak Ł Łabuzek K Bułdak RJ Machnik G Bołdys A Okopień B. Exenatide (a GLP-1 agonist) improves the antioxidative potential of *in vitro* cultured human monocytes/macrophages. Naunyn Schmiedebergs Arch Pharmacol. (2015) 388:905–19. doi: 10.1007/s00210-015-1124-3 PMC4537507 25980358

[40] Ishibashi Y Matsui T Takeuchi M Yamagishi S. Glucagon-like peptide-1 (GLP-1) inhibits advanced glycation end product (AGE)-induced up-regulation of VCAM-1 mRNA levels in endothelial cells by suppressing AGE receptor (RAGE) expression. Biochem Biophys Res Commun. (2010) 391:1405–8. doi: 10.1016/j.bbrc.2009.12.075 20026306

[41] Wang Y Parlevliet ET Geerling JJ van der Tuin SJ Zhang H Bieghs V. Exendin-4 decreases liver inflammation and atherosclerosis development simultaneously by reducing macrophage infiltration. Br J Pharmacol. (2014) 171:723–34. doi: 10.1111/bph.12490 PMC3969084 24490861

[42] Kang HM Kang Y Chun HJ Jeong JW Park C. Evaluation of the *in vitro* and *in vivo* angiogenic effects of exendin-4. Biochem Biophys Res Commun. (2013) 434:150–4. doi: 10.1016/j.bbrc.2013.03.053 23541581

[43] Aronis KN Chamberland JP Mantzoros CS. GLP-1 promotes angiogenesis in human endothelial cells in a dose-dependent manner, through the Akt, Src and PKC pathways. Metabolism. (2013) 62:1279–86. doi: 10.1016/j.metabol.2013.04.010 PMC3755020 23684008

[44] Roan JN Cheng HN Young CC Lee CJ Yeh ML Luo CY. Exendin-4, a glucagon-like peptide-1 analogue, accelerates diabetic wound healing. J Surg Res. (2017) 208:93–103. doi: 10.1016/j.jss.2016.09.024 27993221

[45] Salerno L Floresta G Ciaffaglione V Gentile D Margani F Turnaturi R. Progress in the development of selective heme oxygenase-1 inhibitors and their potential therapeutic application. Eur J Med Chem. (2019) 167:439–53. doi: 10.1016/j.ejmech.2019.02.027 30784878

[46] Seo E Lim JS Jun JB Choi W Hong IS Jun HS. Exendin-4 in combination with adipose-derived stem cells promotes angiogenesis and improves diabetic wound healing. J Transl Med. (2017) 15:35. doi: 10.1186/s12967-017-1145-4 28202074 PMC5311833

[47] Yu M Huang J Zhu T Lu J Liu J Li X. Liraglutide-loaded PLGA/gelatin electrospun nanofibrous mats promote angiogenesis to accelerate diabetic wound healing via the modulation of miR-29b-3p. Biomater Sci. (2020) 8:4225–38. doi: 10.1039/d0bm00442a 32578587

[48] Huang H Wang L Qian F Chen X Zhu H Yang M. Liraglutide via activation of AMP-activated protein kinase-hypoxia inducible factor-1α-heme oxygenase-1 signaling promotes wound healing by preventing endothelial dysfunction in diabetic mice. Front Physiol. (2021) 12:660263. doi: 10.3389/fphys.2021.660263 34483951 PMC8415222

[49] Wolak M Staszewska T Juszczak M Gałdyszyńska M Bojanowska E. Anti-inflammatory and pro-healing impacts of exendin-4 treatment in Zucker diabetic rats: Effects on skin wound fibroblasts. Eur J Pharmacol. (2019) 842:262–9. doi: 10.1016/j.ejphar.2018.10.053 30391742

[50] Nagae K Uchi H Morino-Koga S Tanaka Y Oda M Furue M. Glucagon-like peptide-1 analogue liraglutide facilitates wound healing by activating PI3K/Akt pathway in keratinocytes. Diabetes Res Clin Pract. (2018) 146:155–61. doi: 10.1016/j.diabres.2018.10.013 30367901

[51] Dhatariya K Bain SC Buse JB Simpson R Tarnow L Kaltoft MS. The impact of liraglutide on diabetes-related foot ulceration and associated complications in patients with type 2 diabetes at high risk for cardiovascular events: results from the LEADER trial. Diabetes Care. (2018) 41:2229–35. doi: 10.2337/dc18-1094 PMC6150424 30072400

[52] Schäfer Z Mathisen A Thomsen TR Rossing P Kirketerp-Møller K. Glucagon-like peptide-1 treatment reduces the risk of diabetes-type 2 related amputations: A cohort study in Denmark. Diabetes Res Clin Pract. (2023) 202:110799. doi: 10.1016/j.diabres.2023.110799 37391034

[53] Werkman NCC Driessen JHM Stehouwer CDA Vestergaard P Schaper NC van den Bergh JP. The use of sodium-glucose co-transporter-2 inhibitors or glucagon-like peptide-1 receptor agonists versus sulfonylureas and the risk of lower limb amputations: a nation-wide cohort study. Cardiovasc Diabetol. (2023) 22:160. doi: 10.1186/s12933-023-01897-2 37386427 PMC10311702

[54] Lin DS Lee JK Chen WJ. Major adverse cardiovascular and limb events in patients with diabetes treated with GLP-1 receptor agonists vs DPP-4 inhibitors. Diabetologia. (2021) 64:1949–62. doi: 10.1007/s00125-021-05497-1 34195865

[55] Lin DS Yu AL Lo HY Lien CW Lee JK Chen WJ. Major adverse cardiovascular and limb events in people with diabetes treated with GLP-1 receptor agonists vs SGLT2 inhibitors. Diabetologia. (2022) 65:2032–43. doi: 10.1007/s00125-022-05772-9 35945333

[56] Tahrani AA Barnett AH Bailey CJ. SGLT inhibitors in management of diabetes. Lancet Diabetes Endocrinol. (2013) 1:140–51. doi: 10.1016/s2213-8587(13)70050-0 24622320

[57] Abdul-Ghani MA Norton L Defronzo RA. Role of sodium-glucose cotransporter 2 (SGLT 2) inhibitors in the treatment of type 2 diabetes. Endocr Rev. (2011) 32:515–31. doi: 10.1210/er.2010-0029 21606218

[58] Scheen AJ Paquot N. Metabolic effects of SGLT-2 inhibitors beyond increased glucosuria: A review of the clinical evidence. Diabetes Metab. (2014) 40:S4–s11. doi: 10.1016/s1262-3636(14)72689-8 25554070

[59] Scheen AJ. Pharmacokinetics, pharmacodynamics and clinical use of SGLT2 inhibitors in patients with type 2 diabetes mellitus and chronic kidney disease. Clin Pharmacokinet. (2015) 54:691–708. doi: 10.1007/s40262-015-0264-4 25805666

[60] Scheen AJ. Pharmacodynamics, efficacy and safety of sodium-glucose co-transporter type 2 (SGLT2) inhibitors for the treatment of type 2 diabetes mellitus. Drugs. (2015) 75:33–59. doi: 10.1007/s40265-014-0337-y 25488697

[61] Scheen AJ. SGLT2 inhibitors: benefit/risk balance. Curr Diabetes Rep. (2016) 16:92. doi: 10.1007/s11892-016-0789-4 27541294

[62] Oguntibeju OO. Type 2 diabetes mellitus, oxidative stress and inflammation: examining the links. Int J Physiol Pathophysiol Pharmacol. (2019) 11:45–63. 31333808 PMC6628012

[63] Miceli G Basso MG Pennacchio AR Cocciola E Pintus C Cuffaro M. The potential impact of SGLT2-I in diabetic foot prevention: promising pathophysiologic implications, state of the art, and future perspectives-A narrative review. Medicina (Kaunas). (2024) 60(11). doi: 10.3390/medicina60111796 PMC11596194 39596981

[64] Piaggesi A. Research development in the pathogenesis of neuropathic diabetic foot ulceration. Curr Diabetes Rep. (2004) 4:419–23. doi: 10.1007/s11892-004-0050-4 15539005

[65] Mueller MJ Minor SD Diamond JE Blair VP 3rd. Relationship of foot deformity to ulcer location in patients with diabetes mellitus. Phys Ther. (1990) 70:356–62. doi: 10.1093/ptj/70.6.356 2345779

[66] Abdelkader NF Elbaset MA Moustafa PE Ibrahim SM. Empagliflozin mitigates type 2 diabetes-associated peripheral neuropathy: a glucose-independent effect through AMPK signaling. Arch Pharm Res. (2022) 45:475–93. doi: 10.1007/s12272-022-01391-5 PMC9325846 35767208

[67] Ishibashi F Kosaka A Tavakoli M. Sodium glucose cotransporter-2 inhibitor protects against diabetic neuropathy and nephropathy in modestly controlled type 2 diabetes: follow-up study. Front Endocrinol (Lausanne). (2022) 13:864332. doi: 10.3389/fendo.2022.864332 35784562 PMC9247156

[68] Kandeel M. The outcomes of sodium-glucose co-transporter 2 inhibitors (SGLT2I) on diabetes-associated neuropathy: A systematic review and meta-analysis. Front Pharmacol. (2022) 13:926717. doi: 10.3389/fphar.2022.926717 35899123 PMC9310020

[69] Mohsin F Javaid S Tariq M Mustafa M. Molecular immunological mechanisms of impaired wound healing in diabetic foot ulcers (DFU), current therapeutic strategies and future directions. Int Immunopharmacol. (2024) 139:112713. doi: 10.1016/j.intimp.2024.112713 39047451

[70] Xu L Nagata N Nagashimada M Zhuge F Ni Y Chen G. SGLT2 inhibition by empagliflozin promotes fat utilization and browning and attenuates inflammation and insulin resistance by polarizing M2 macrophages in diet-induced obese mice. EBioMedicine. (2017) 20:137–49. doi: 10.1016/j.ebiom.2017.05.028 PMC5478253 28579299

[71] Qin J Liu Q Liu A Leng S Wang S Li C. Empagliflozin modulates CD4(+) T-cell differentiation via metabolic reprogramming in immune thrombocytopenia. Br J Haematol. (2022) 198:765–75. doi: 10.1111/bjh.18293 35675486

[72] Jenkins BJ Blagih J Ponce-Garcia FM Canavan M Gudgeon N Eastham S. Canagliflozin impairs T cell effector function via metabolic suppression in autoimmunity. Cell Metab. (2023) 35:1132–1146.e1139. doi: 10.1016/j.cmet.2023.05.001 37230079

[73] Fernández−Balsells MM Sojo-Vega L Ricart-Engel W. Canagliflozin and cardiovascular and renal events in type 2 diabetes. N Engl J Med. (2017) 377:2098. doi: 10.1056/NEJMc1712572 29182249

[74] Lin C Zhu X Cai X Yang W Lv F Nie L. SGLT2 inhibitors and lower limb complications: an updated meta-analysis. Cardiovasc Diabetol. (2021) 20:91. doi: 10.1186/s12933-021-01276-9 33910574 PMC8082772

[75] Kaneko M Narukawa M. Effects of sodium-glucose cotransporter 2 inhibitors on amputation, bone fracture, and cardiovascular outcomes in patients with type 2 diabetes mellitus using an alternative measure to the hazard ratio. Clin Drug Investig. (2019) 39:179–86. doi: 10.1007/s40261-018-0731-4 30506378

[76] Du Y Bai L Fan B Ding H Ding H Hou L. Effect of SGLT2 inhibitors versus DPP4 inhibitors or GLP-1 agonists on diabetic foot-related extremity amputation in patients with T2DM: A meta-analysis. Prim Care Diabetes. (2022) 16:156–61. doi: 10.1016/j.pcd.2021.12.007 34930687

[77] Ryan PB Buse JB Schuemie MJ DeFalco F Yuan Z Stang PE. Comparative effectiveness of canagliflozin, SGLT2 inhibitors and non-SGLT2 inhibitors on the risk of hospitalization for heart failure and amputation in patients with type 2 diabetes mellitus: A real-world meta-analysis of 4 observational databases (OBSERVE-4D). Diabetes Obes Metab. (2018) 20:2585–97. doi: 10.1111/dom.13424 PMC6220807 29938883

[78] Dorsey-Treviño EG González-González JG Alvarez-Villalobos N González-Nava V Contreras-Garza BM Díaz González-Colmenero A. Sodium-glucose cotransporter 2 (SGLT-2) inhibitors and microvascular outcomes in patients with type 2 diabetes: systematic review and meta-analysis. J Endocrinol Invest. (2020) 43:289–304. doi: 10.1007/s40618-019-01103-9 31489568

[79] Chang HY Singh S Mansour O Baksh S Alexander GC. Association between sodium-glucose cotransporter 2 inhibitors and lower extremity amputation among patients with type 2 diabetes. JAMA Intern Med. (2018) 178:1190–8. doi: 10.1001/jamainternmed.2018.3034 PMC6142968 30105373

[80] Lee YC Dong YH Yang WS Wu LC Lin JW Chang CH. Risk of major adverse limb events in patients with type 2 diabetes mellitus receiving sodium glucose cotransporter 2 inhibitors and glucagon-like peptide-1 receptor agonists: A population-based retrospective cohort study. Front Pharmacol. (2022) 13:869804. doi: 10.3389/fphar.2022.869804 36176438 PMC9513310

[81] Chang HY Chou YY Tang W Chang GM Hsieh CF Singh S. Association of antidiabetic therapies with lower extremity amputation, mortality and healthcare cost from a nationwide retrospective cohort study in Taiwan. Sci Rep. (2021) 11:7000. doi: 10.1038/s41598-021-86516-4 33772082 PMC7997872

[82] Fralick M Kim SC Schneeweiss S Everett BM Glynn RJ Patorno E. Risk of amputation with canagliflozin across categories of age and cardiovascular risk in three US nationwide databases: cohort study. BMJ. (2020) 370:m2812. doi: 10.1136/bmj.m2812 32843476 PMC7445737

[83] Ueda P Svanström H Melbye M Eliasson B Svensson AM Franzén S. Sodium glucose cotransporter 2 inhibitors and risk of serious adverse events: nationwide register based cohort study. BMJ. (2018) 363:k4365. doi: 10.1136/bmj.k4365 30429124 PMC6233755

[84] Rodionov RN Peters F Marschall U L’Hoest H Jarzebska N Behrendt CA. Initiation of SGLT2 inhibitors and the risk of lower extremity minor and major amputation in patients with type 2 diabetes and peripheral arterial disease: A health claims data analysis. Eur J Vasc Endovasc Surg. (2021) 62:981–90. doi: 10.1016/j.ejvs.2021.09.031 34782230

[85] Fu EL D’Andrea E Wexler DJ Patorno E Paik JM. Safety of sodium-glucose cotransporter-2 inhibitors in patients with CKD and type 2 diabetes: population-based US cohort study. Clin J Am Soc Nephrol. (2023) 18:592–601. doi: 10.2215/cjn.0000000000000115 36827225 PMC10278835

[86] Werkman NCC Nielen JTH van den Bergh JPW Ejskjaer N Røikjer J Schaper NC. Use of sodium-glucose co-transporter-2-inhibitors (SGLT2-is) and risk of lower limb amputation. Curr Drug Saf. (2021) 16:62–72. doi: 10.2174/1574886315666200805103053 32767909

[87] Dawwas GK Smith SM Park H. Cardiovascular outcomes of sodium glucose cotransporter-2 inhibitors in patients with type 2 diabetes. Diabetes Obes Metab. (2019) 21:28–36. doi: 10.1111/dom.13477 30039524

[88] D’Andrea E Wexler DJ Kim SC Paik JM Alt E Patorno E. Comparing effectiveness and safety of SGLT2 inhibitors vs DPP-4 inhibitors in patients with type 2 diabetes and varying baseline hbA1c levels. JAMA Intern Med. (2023) 183:242–54. doi: 10.1001/jamainternmed.2022.6664 PMC9989905 36745425

[89] Patorno E Pawar A Wexler DJ Glynn RJ Bessette LG Paik JM. Effectiveness and safety of empagliflozin in routine care patients: Results from the EMPagliflozin compaRative effectIveness and SafEty (EMPRISE) study. Diabetes Obes Metab. (2022) 24:442–54. doi: 10.1111/dom.14593 PMC8939295 34729891

[90] Lin YH Lin CH Lin YC Huang YY Tai AS Fu SC. Sodium-glucose cotransporter 2 inhibitors reduce the risk of hospitalization for heart failure and amputation rate compared with incretin-based therapy in patients with diabetic foot disease: A nationwide population-based study. Endocr Pract. (2024) 30:424–30. doi: 10.1016/j.eprac.2024.01.016 38325629

[91] Fadini GP Avogaro A. SGLT2 inhibitors and amputations in the US FDA Adverse Event Reporting System. Lancet Diabetes Endocrinol. (2017) 5:680–1. doi: 10.1016/s2213-8587(17)30257-7 28733172

[92] Potier L Roussel R Velho G Saulnier PJ Bumbu A Matar O. Lower limb events in individuals with type 2 diabetes: evidence for an increased risk associated with diuretic use. Diabetologia. (2019) 62:939–47. doi: 10.1007/s00125-019-4835-z 30809716

[93] Xiao F Rui S Zhang X Ma Y Wu X Hao W. Accelerating diabetic wound healing with Ramulus Mori (Sangzhi) alkaloids via NRF2/HO-1/eNOS pathway. Phytomedicine. (2024) 134:155990. doi: 10.1016/j.phymed.2024.155990 39243750

